# ATBS1-INTERACTING FACTOR 2 Negatively Modulates Pollen Production and Seed Formation in *Arabidopsis*

**DOI:** 10.3389/fpls.2021.704958

**Published:** 2021-07-27

**Authors:** Yoon Kim, Sun-Ho Kim, Dong-Min Shin, Soo-Hwan Kim

**Affiliations:** Division of Biological Science and Technology, Yonsei University, Wonju, South Korea

**Keywords:** auxin, ATBS1-INTERACTING FACTOR 2, BRASSINOSTEROID-INSENSITIVE 2, brassinosteroid, BRASSINAZOLE RESISTANT 1, pollen, seed

## Abstract

ATBS1-INTERACTING FACTOR 2 (AIF2) is a non-DNA-binding basic-helix-loop-helix (bHLH) transcription factor. Here, we demonstrate that AIF2 negatively modulates brassinosteroid (BR)-induced, BRASSINAZOLE RESISTANT 1 (BZR1)-mediated pollen and seed formation. *AIF2*-overexpressing *Arabidopsis* plants (AIF2ox) showed defective pollen grains and seed production while two *AIF2* knockout mutants, *aif2-1* and *aif2-1*/*aif4-1*, displayed opposite phenotypes. Genes encoding BZR1-regulated positive factors of seed size determination (*SHB1*, *IKU1*, *MINI3*) were suppressed in AIF2ox and genes for negative factors (*AP2* and *ARF2*) were enhanced. Surprisingly, BZR1-regulated pollen genes such as *SPL*, *MS1*, and *TDF1* were aberrantly up-regulated in AIF2ox plants. This stage-independent abnormal expression may lead to a retarded and defective progression of microsporogenesis, producing abnormal tetrad microspores and pollen grains with less-effective pollen tube germination. Auxin plays important roles in proper development of flower and seeds: genes for auxin biosynthesis such as *TCP*s and *YUCCA*s as well as for positive auxin signalling such as *ARF*s were suppressed in AIF2ox flowers. Moreover, lipid biosynthesis- and sucrose transport-related genes were repressed, resulting in impaired starch accumulation. Contrarily, sucrose and BR repressed ectopic accumulation of AIF2, thereby increasing silique length and the number of seeds. Taken together, we propose that AIF2 is negatively involved in pollen development and seed formation, and that sucrose- and BR-induced repression of AIF2 positively promotes pollen production and seed formation in *Arabidopsis*.

## Introduction

Seed development and seed size determination in plants are complicated processes controlled by diverse hormones and downstream transcription factors (Sun X. et al., 2010; Li and Li, 2016). Seeds comprise three genetically distinctive structures: the embryo giving rise to the seedling, the endosperm providing nutrients for the embryo, and the seed coat enclosing the embryo and endosperm. The endosperm arises from the central cell and constitutes the major volume of the mature seed. In *Arabidopsis* after fertilisation, rapid proliferation and expansion of the endosperm occurs to generate a large and multinucleated cell or syncytium until the embryo reaches the heart stage and results in a large increase in seed size or volume of the seed cavity (Sun X. et al., 2010). Several factors have been shown to control seed size by regulating endosperm growth (Li and Li, 2016). Loss-of-function mutations of *HAIKU* (*IKU*) and the WRKY transcription factor *MINI-SEED 3* (*MINI3*) caused precocious cellularisation of the syncytial endosperm resulting in the reduction in endosperm size and embryo proliferation (Garcia et al., 2003; Luo et al., 2005; Wang et al., 2010). The recruitment of SHORT HYPOCOTYL UNDER BLUE1 (SHB1) by MINI3 to its own and *IKU2* promoters upregulated their expression (Zhou et al., 2009). *Arabidopsis* APETALA2 (AP2) encodes a plant transcription factor having the AP domain that is negatively involved in regulation of seed size and numbers (Ohto et al., 2009). *ap2* seeds underwent an early expanded growth period that was associated with delayed endosperm cellularisation and outgrowth of the endosperm central vacuole, resulting in an increase in embryo cell number and size, enlarged embryo sac, and large seeds with increased total protein and oil content (Jofuku et al., 2005; Ohto et al., 2009). Additionally, proteins involved in ubiquitin–proteasome pathways, G-protein signalling, mitogen-activated protein kinase signalling, and epigenetic regulation and paternal imprinting were substantially involved in the control of seed size and numbers (Sun X. et al., 2010; Li and Li, 2016; Li N. et al., 2019).

Plant hormones are closely involved in the regulation of reproduction, embryogenesis, and determination of seed size and yields. Auxin signalling was closely linked to endosperm development, embryo polarity, and patterning (Figueiredo and Köhler, 2018). Embryo sacs of plants selectively silenced for AUXIN RESPONSIVE FACTORs (ARFs) exhibited identity defects at the micropylar pole, and the pollen grains were morphologically aberrant and unviable (Liu et al., 2018). In addition, a loss-of-function mutant of *ARF2*, initially identified as *mmt* mutant, showed extra cell division in the integuments surrounding the ovule, leading to the formation of enlarged seed coats and seed size (Schruff et al., 2006). ABSCISIC ACID-INSENSITIVE5 (ABI5)-mediated abscisic acid (ABA) signalling pathways were negatively involved in the early stage of seed development by suppressing *SHB1* expression; thus, an ABA biosynthesis-deficient mutant, *aba2-1*, produced seeds with increased size, mass, and embryo cell number (Cheng et al., 2014). YODA (YDA) is a mitogen-activated protein kinase, and YDA and ETHYLENE-INSENSITIVE3 (EIN3) were integral to a sugar-mediated metabolism cascade regulating seed mass by maternally controlling embryo and seed sizes (Meng et al., 2018). Transcriptome analysis of the early stage of proliferating endosperm revealed that cytokinin signalling-related genes were significantly enriched (Day et al., 2008). Indeed, triple loss-of-function mutants of cytokinin receptors, *ahk2 ahk3 cre1*, produced enlarged but fewer seeds per silique, and this increase in seed size was correlated with an increase in the size of the mutant embryo (Riefler et al., 2006).

Brassinosteroids (BRs) are plant steroid hormones that play crucial roles in plant growth and development via extensive signal integration through direct interactions with numerous signalling pathways (Kim and Russinova, 2020). Upon binding of BRs to BRASSINOSTEROID-INSENSITIVE 1 (BRI1), the activation of BRI1 and BRI1-ASSOCIATED RECEPTOR KINASE 1 (BAK1) complex and the subsequent phosphorylation of BRASSINOSTEROID SIGNALLING KINASE (BSK) initiated a signalling cascade, relaying the membrane surface signal to the nucleus to activate the positively acting transcription factors BRASSINAZOLE RESISTANT 1/BRI1 EMS SUPPRESSOR 2 (BZR1/BES2) and BZR2/BES1 (He et al., 2005; Sun Y. et al., 2010). In the absence of BRs, their growth-promoting pathways were negatively balanced through GSK3/SHAGGY-LIKE BRASSINOSTEROID-INSENSITIVE 2 (ATSK21/BIN2)-mediated BZR1/BES1 degradation (He et al., 2002) and the antagonistic BIN2-driven increase in ATBS1-INTERACTING FACTOR 2 (AIF2) stability, an atypical non-DNA-binding bHLH transcription factor acting as a negative regulator of BR-induced growth promotion (Kim et al., 2017). Other AIF2 homologues such as AIF1, AIF3, and AIF4 were also identified with their high amino acid sequence similarity, and these AIFs were functionally redundant in inhibiting plant growth (Wang et al., 2009; Ikeda et al., 2013; Kim et al., 2017).

Environmental and endogenous stimuli affecting the timing and duration of reproductive phase can significantly impact seed yields (Shirley et al., 2019). In this regard, BRs control diverse aspects of floral organ formation, embryo and seed development, and seed size determination. For example, BSK family proteins contributed to early embryonic patterning, and *bsk1bsk2* double loss-of-function mutants exhibited reduced zygote cell growth, which resulted in a small basal cell followed by a small suspensor cell (Neu et al., 2019). Moreover, BR biosynthesis- (*cpd*) or signalling-defective (*bri1-116*, *bin2-1*) mutants had greatly reduced number of pollen grains and were defective in pollen release and exine pattern formation (Ye et al., 2010). The same study showed that BES1, a positive transcription activator for BR signalling pathways, directly bound to promoter regions of genes (*SPL*/*NZZ*, *TDF1*, *AM5*, *MS1*, and *MS2*) encoding proteins essential for anther and pollen development (Chen W. et al., 2019). BZR1 (a BES1 homologue) family transcription factors were also involved in the regulation of anther development, in a BRI1-independent manner, by upregulating *SPOROCYTELESS* (*SPL*) and its upstream activator *AGAMOUS* (*AG*) that were required for the initiation of archesporial cells (Chen L. G. et al., 2019). Furthermore, BZR1-mediated BR signalling pathways positively influenced seed numbers by regulating the expression of genes (*HLL*, *ANT*, *AP2, INO*) that were involved in development of ovule and ovule integument (Huang et al., 2013; Jia et al., 2020). BZR1 also directly bound to and activated positive regulators of seed development (*SHB1*, *MINI3*, and *IKU2*) and repressed negative regulators of seed size (*AP2* and *ARF2*) (Jiang et al., 2013). In this study, we demonstrate that AIF2 is negatively involved in pollen development and seed formation, and that sucrose- and BR-induced repression of AIF2 bHLH transcription factor positively controls pollen production and seed size/numbers in *Arabidopsis*.

## Materials and Methods

### Plant Material and Growth Conditions

Wild-type *Arabidopsis thaliana* (Col-0 and WS), an *AIF2* T-DNA knockout mutant (*aif2-1*, CS811403), *aif2-1*/*aif4-1* double knockout mutant, *AIF2* overexpressing *p35S:AIF2-EGFP* transgenic plants (AIF2ox, Kim et al., 2017), a *BIN2* triple knockout mutant (*bin2*KO, *bin2bil1bil2*; Yan et al., 2009), a *BIN2* gain-of-function mutant *bin2-1* (Peng et al., 2008), *arf2-7* (Okushima et al., 2005), a *BZR1* gain-of-function mutant *bzr1-1D* (Wang et al., 2002), and a BZR1 dominant negative mutant *bzr1-1DdEAR* (Oh et al., 2014) were used for phenotype analysis and generation of transgenic plants. A native promoter-driven reporter plant, *pAIF2:AIF2-GUS*, was used to observe *AIF2* expression and localisation. Seeds were surfaced-sterilised with 95% ethanol for 10 min and cold-treated in the dark at 4°C for 72 h. These sterilised seeds were then sown in pots containing Sunshine No. 5 soil (Polysciences, United States) and grown in a growth room operating under a 16 h light (100–150 μmol m^–2^ s^–1^) and 8 h dark cycle at 23–25°C.

### Generation of Transgenic Plants With Different Mutant Backgrounds

For generation of transgenic plants ectopically expressing variants of AIF2-EGFP, cDNAs encoding either the full-length (AIF2ox) or C-terminus truncated forms of AIF2 (AIF2dC) were amplified using primers listed (Supplementary Table 1) and inserted upstream of the myc-tag EGFP-expressing pB7FWG2 (Karimi et al., 2002) binary vectors. Subsequently, *Agrobacterium* cultures carrying each construct were used to transform Col-0, *aif2-1*, *bzr1-1D*, *bin2-1*, and *bin2bil1bil2* genetic lines, generating plants designated as *p35S:AIF2FL-EGFP*/Col-0 (AIF2ox), *p35S::AIF2dC-EGFP/*Col-0 (AIF2dC), *p35S:AIF2FL-EGFP*/*aif2-1*(AIF2ox/*aif2-1*), *p35S:AIF2dC-EGFP*/*aif2-1*(AIF2dC/*aif2-1*), *p35S:AIF2FL-EGFP*/*bzr1-1D* (AIF2ox/*bzr1-1D*), *p35S:AIF2dC-EGFP*/*bzr1-1D* (AIF2dC/*bzr1-1D*), *p35S:AIF2FL-EGFP*/*bin2bil1bil2* (AIF2ox/*bin2bil1bil2*), and *p35S:AIF2dC-EGFP*/*bin2-1* (AIF2dC/*bin2-1*). Pollen grains of AIF2ox or *bzr1-1DdEAR* were crossed to a stigma of *arf2-7* or *aif2-1* plants to produce the AIF2ox/*arf2-7* and the *bzr1-1DdEAR*/*aif2-1* transgenic plants, respectively.

### Generation of a*if2-1/aif4-1* Double Knockout Plants

The CRISPR-Cas9 system was used as described previously (Kim et al., 2016). Briefly, guide RNA sequences targetting the exon of *AIF4* (At1g09250) gene were designed using the guide RNA(gRNA) design tool (Concordet and Haeussler, 2018)^1^ as follows: 5′-GATTGAACTCGTCTCCGGCGCGGCG-3′ and 5′-AAACCGC CGCGCCGGAGACGAGTTC-3. The complementary gRNA was then inserted into pHAtC vector, and the resulting construct was transformed into *aif2-1* to generate the *aif2-1*/*aif4-1* double knockout mutant. A deletion of guanine at base pair position 249 starting from the initiator ATG was confirmed by performing DNA sequencing for the PCR-amplified *AIF4* gene.

### Total RNA Isolation and qRT-PCR Analysis

Total RNAs were extracted using a plant RNA extraction kit (Intron Biotechnology, South Korea) from flowers at different floral stages, siliques with developing seeds, or siliques isolated from *in vitro*-cultured flowers. To examine semi-quantitative RNA expression, the first-strand cDNA was synthesised using a ReverTra Ace qPCR RT Master Mix kit (Toyobo) according to the manufacturer’s instructions. Quantitative real-time RT-PCR (qRT-PCR) was performed by the SYBR green method using the Applied Biosystems Step One Plus System (Applied Biosystems, United States) with appropriate primers (Supplementary Table 2). The conditions for PCR amplification were as follows: 1 cycle of 95°C for 10 min; 40 cycles of 95°C for 15 s, 60°C for 30 s, and 72°C for 30 s. Expression of each transcript was normalised against the amount of *UBC1* control in each sample. Three biological replicates were included in each experiment, and expression in each replicate was measured three times.

### Protein Isolation and Western Blot Analysis

To examine expression of AIF2-EGFP in AIF2ox transgenic plants, total proteins were extracted from *in vitro*-cultured flowers using a homogenisation buffer (125 mM Tris-Cl, 4% sodium dodecyl sulphate, 2% β-mercaptoethanol, 1 mM phenylmethylsulfonyl, pH 7.9) and size-fractionated on 12% SDS-PAGE. Fractioned total proteins were then transferred onto a nitrocellulose membrane (Whatman, Germany) and probed against anti-GFP rabbit polyclonal antibodies (Santa Cruz Biotechnology, United States) in 5% milk/TBST (50 mM Tris-acetate, 150 mM NaCl, 0.05% Tween 20, pH 7.6). Goat anti-rabbit HRP-conjugated secondary antibody (Abcam, United Kingdom) was used to quantify the AIF2-EGFP protein. Peroxidase activity was detected using an ECL solution (Thermo Fisher Scientific Inc., United States) according to the manufacturer’s instructions.

### Histochemical Staining and Microscopic Observation

For pollen grain staining, anthers were removed from newly opening flowers and stained with Alexander’s solution for 8 h at 50°C (Peterson et al., 2010), mPS-PI solution for 2 h at room temperature (Truernit et al., 2008), or pollen isolation solution containing 5μg/ml DAPI. Stained anthers or pollens were then observed using either a differential interference contrast (DIC)-equipped fluorescence microscope (Olympus BX60, Japan) or a Meta NLO-UV confocal laser scanning microscope (Zeiss LSM510, Germany).

To examine *in vivo* pollen tube growth, pistils were hand-pollinated with pollen grains of the same flower. The pollinated pistils were then fixed with 25% acetic acid at different times (h) after pollination, hydrated with an ethanol series, and softened with NaOH. Pollen tubes were then stained with aniline blue following a previously reported method (Mori et al., 2006) and their growth was examined using the Zeiss confocal microscope. For *in vitro* pollen tube growth assay, pollen grains were collected from 10–20 freshly opened flowers and grown on a solid germination medium (Boavida and McCormick, 2007) for 6 h in the dark at room temperature. Pollen tubes were photographed using a camera connected to the DIC-equipped Olympus microscope and their lengths were measured using Image J.^2^

To examine AIF2 expression *in planta*, opened flowers of *pAIF2:AIF2-GUS* transgenic plants were collected and fixed in 90% acetone for 20 min on ice. Staining and detection of GUS activity were performed according to the method described by Jefferson (1987). The stained floral organs were observed under the DIC-equipped Olympus microscope.

For starch staining of developing seeds, pistils were hand-pollinated with pollen grains of the same flower. Developing siliques were then collected at different days after pollination (DAP), placed in fixing solution containing 10% acetic acid and 90% ethanol (v/v), and incubated overnight in a water bath at 60°C followed by washing with 70% ethanol. Siliques were then stained in Lugol’s iodine solution for 5 min and observed under a DIC-equipped Olympus microscope.

### Seed Clearing and Imaging Analysis

For determination of embryo developmental stages, siliques were fixed overnight in solution containing 10% acetic acid and 90% ethanol (v/v) and washed twice sequentially with 90% and 70% ethanol. Siliques were then cleared overnight with chloral hydrate solution (Yadegari et al., 1994). These cleared seeds from siliques were mounted in clearing solution for observation under the Olympus microscope. Afterward, the embryo area and the rest of the integument-surrounded area were measured using Image J.

### *In vitro* Flower Culture

Flowers in an early stage of seed development (between DAP3.5 to DAP4) were cut and immediately transferred to 30% ethanol for 3 min. These sterilised flowers were placed in half-strength solid MS media containing brassinolide (BL, 10^–9^ M) supplemented with or without 3% sucrose (w/v). These flowers were then cultured for 9 days in a growth chamber operating under a 16 h light (100–150 μmol m^–2^ s^–1^) and 8 h dark cycle at 23–25°C. Siliques were collected from *in vitro*-cultured flowers to examine their phenotypes.

### Measurement and Statistical Analysis

Over 100 siliques or flowers were collected from 30–40 plants and used for each experiment. All experiments were conducted in triplicate at a minimum, and the data were statistically analysed using the Student’s *t*-test.

## Results

### Overexpression of AIF2 Resulted in Defective Formation of Pollen Grains and Reduced Seed Production

Previously, we demonstrated that AIF2 were negatively involved in BR-induced growth regulation (Kim et al., 2017); nonetheless, its roles in the development of other organs are unknown. As an initial step to elucidate the roles of AIF2 in pollen and seed development, we first investigated silique phenotype of AIF2ox transgenic plants (*p35S*:*AIF2-EGFP*/Col-0). Three independent transgenic lines (AIF2ox-1 to AIF2ox-3) differentially expressed AIF2 protein, ranged from high to low levels compared with the Col-0 plants (AIF2ox-1 to AIF2ox-3, respectively) and showed retarded growth phenotypes as previously reported (Supplementary Figures 1A,B; Kim et al., 2017). Interestingly, their siliques were smaller in AIF2ox plants, and their reduction in silique length at floral stage 17 was inversely correlated with the abundance of AIF2 proteins: for instance, AIF2ox-1 showed the most severely retarded silique phenotype (Supplementary Figures 1C,D). Hereafter, we took AIF2ox-1 line for further analysis of pollen, embryo, and seed phenotypes.

**FIGURE 1:**
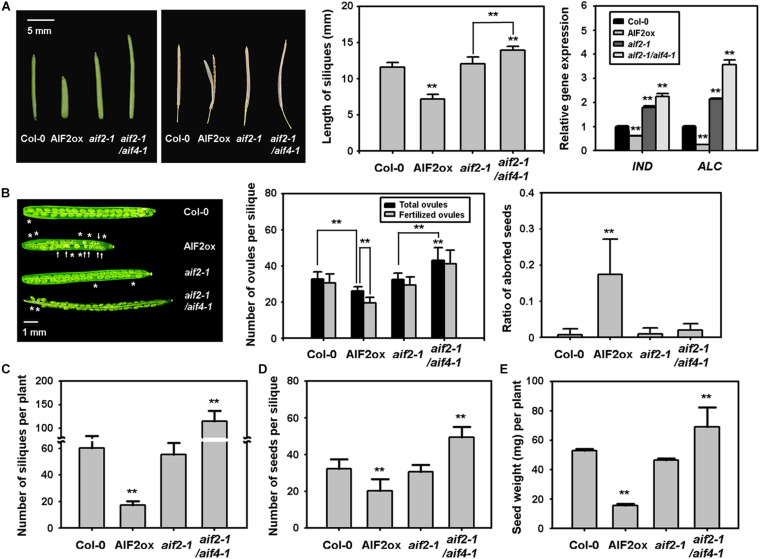
Defective silique phenotypes of *AIF2*-overexpressing *p35S:AIF2-EGFP* transgenic plants (AIF2ox). **(A)** Silique with shorter and premature dehiscence phenotypes. The picture on the left shows representative shorter and unfused gynoecia silique phenotypes. Siliques from opened flowers were measured for their length (middle) and transcript expression of genes involved in valve margin development of the carpel (right). Relative expression of each gene is normalised as a ratio of the Col-0 plants, which was set to 1. **(B)** Reduced ovule development and aborted seeds. The picture on the left shows regions lacking ovule development (*) and aborted fertilised eggs (arrow). Total ovules presented in the middle are calculated by the sum of visibly developed ovules and invisible empty ovule area. Fertilised ovules indicate the visible ovules. The ratio of aborted seeds on the right represents the ratio of seeds with defective phenotype (such as smaller or white) to total visible seeds. **(C–E)** Number of siliques **(C)**, seeds **(D)**, and total seed weight **(E)** was measured from more than 100 siliques collected from 30–40 plants. Bar graphs represent means ± SD and statistical difference from either the Col-0 control or bracketed samples is indicated by two asterisks (**) at *P* < 0.01.

Disrupting pollen/ovule development, pollination, pollen tube growth, and fertilisation results in a reduced number of seed sets and silique size. We found that *AIF2*-overexpressing transgenic *Arabidopsis* plants produced smaller and frequently unfused siliques (Figure 1A, left and middle panels). Regarding unfused siliques, INDEHISCENT (IND), SPATULA (SPT), and ALCATRAZ (ALC) are bHLH transcription factors required for proper valve margin development and later differentiation of the silique dehiscence zone, allowing seed dispersal (Girin et al., 2011; Groszmann et al., 2011; Kay et al., 2013). Accordingly, we found that *IND* and *ALC* expression was down-regulated in *AIF2-*overexpressing plants, whereas it was upregulated in *aif2-1* and *aif2-1*/*aif4-1* plants (Figure 1A, right panel). In addition to the short and unfused silique phenotypes, the number of ovules in a silique was also greatly reduced in AIF2ox (Figure 1B, left and middle panels), and even the fertilised eggs of AIF2ox plants produced a higher ratio of aborted seeds (Figure 1B, right panel). Consequently, the number of both siliques per plant (Figure 1C) and seeds per silique (Figure 1D) in AIF2ox plants was lower than that in wild-type Col-0, resulting in a significantly reduced total seed weight or productivity in AIF2ox plants (Figure 1E). In contrast, *aif2-1*/*aif4-1* plants displayed opposite silique and seed phenotypes.

**FIGURE 2:**
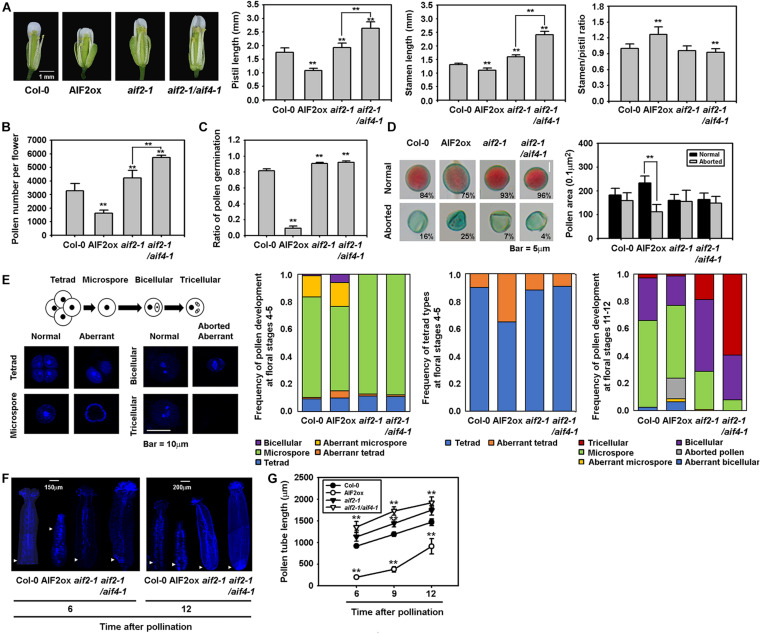
Retarded growth of reproductive organs and degenerated pollen production and germination found in AIF2ox plants. **(A)** Flowers of Col-0, AIF2ox, *aif2-1* and *aif2-1/aif4-1* plants and the lengths of pistils, stamens, and the ratio of stamen to pistil. Number of open flowers examined >30. **(B)** Measurement of pollen numbers counted under a bright microscope without pollen staining. Number of open flowers examined >20. **(C)** *In vitro* pollen germination analysis. The efficiency of germination is represented by the ratio of germinated pollens after 6 h incubation in the germination medium. Number of pollens examined >3,000 taken from 20–25 flowers. **(D)** Frequency and size of the Alexander solution -stained viable pollens (normal) and non-stained aborted pollens. Number of pollens examined >2,000 taken from 15–20 open flowers **(E)** Defective pollen development in AIF2ox flowers. Pollens in different floral stages were stained with DAPI to reveal pollen developmental stages and pollens with intact nuclei (normal), without (aborted) or abnormal nuclei having defects in mitosis and appearance (aberrant). Number of pollens examined >1,000 taken from flowers at stage 4–5 or stage 11-12. **(F,G)** *In vivo* pollen tube growth assay. Arrowhead indicates the end of aniline blue-stained pollen tubes at 6 or 12 h after hand-pollinated self-pollination in the same flowers **(F)** and lengths of pollen tubes at different times were measured after hand-pollination **(G)**. *n* > 15 for each time point was used for analysis. Statistical difference from either the Col-0 control or bracketed samples is indicated by two asterisks (**) at *P* < 0.01.

*Arabidopsis* plants are propagated through self-pollination; thus, the ratio of stamen to pistil length is important for successful pollination. We found that pistils and stamens of AIF2ox plants were shorter than those of Col-0 plants (Figure 2A, 1^*st*^ to 3^*rd*^ panels). Two knockout plants of *AIF2*, *aif2-1* and *aif2-1*/*aif4-1*, had longer pistils and stamen. Nonetheless, the ratio of stamen to pistil was higher in AIF2ox plants (Figure 2A, right panel) indicating that the reduced growth of the stamen or pistil in AIF2ox plants is unlikely to be the cause of the reduced seed production and retarded silique development.

Next, we examined pollen productivity and viability. *AIF2* was specifically expressed in unfertilised ovules and pollen grains of *pAIF2:AIF2-GUS* plants but not in the petal, sepal, stigma, and style (Supplementary Figure 2A). This expression pattern of *AIF2* implies that AIF2 may play a role in male- and female gametophyte development. Similarly, AIF2-EGFP proteins of AIF2ox plants were abundantly expressed in ovules and pollen grains (Supplementary Figures 2B–D). Interestingly, AIF2ox plants contained fewer pollen grains in the anthers, and this reduction in pollen numbers was inversely correlated with the expression levels of AIF2 proteins (Supplementary Figure 3). Consequently, the number of pollens harvested from all anthers from a flower was lower in AIF2ox plants, but slightly higher in *aif2-1* and *aif2-1*/*aif4-1* plants, than in Col-0 plants (Figure 2B). To test pollen viability, we performed *in vitro* pollen germination assay and found that the efficiency of pollen germination was dramatically reduced in AIF2ox plants (Figure 2C). More than 80% of pollen grains in Col-0 and the two *AIF2* knockout plants germinated successfully and initiated pollen tube growth, whereas only 9.2% of pollen germinated in AIF2ox plants. This poor germination efficiency may due to, in part, the high frequency of aborted and smaller pollens commonly observed in AIF2ox plants (Figure 2D).

In *Arabidopsis*, microspore mother cell (2N) undergoes a series of meiosis I and meiosis II (microsporogenesis) to produce a tetrad of microspores (N), and each microspore develops into a bicellular pollen containing a vegetative cell and a generative cell and further to tricellular mature pollen having one vegetative cell and two sperm cells (microgametogenesis) (Figure 2E, 1^*st*^ panel). We found that AIF2ox plants produced higher frequency of aberrant tetrad (36% of tetrads), a tetrad microspore having no nucleus or less microspores, in flowers of stage 4—5 (Figure 2E, 2^*nd*^ and 3^*rd*^ panel). These defects may lead an increased ratio of aborted/aberrant microspore and bicellular pollens (Figure 2E, 4^*th*^ panel). In contrast, the ratio of normal microspore, bicellular and tricellular pollens at floral stage 11—12 was decreased. These results imply that AIF2ox plants underwent a defective microsporogenesis, thus produced less mature and viable pollens. Notably, it seemed that male gametophytogenesis progressed faster in *aif2-1* and *aif2-1*/*aif4-1* than in Col-0 plants (Figure 2E, 4^*th*^ panel). Nonetheless, they had a similar ratio of normal microspore, bicellular and tricellular pollens in total. To further test pollen activity, we manually self-pollinated stigmas of test plants and measured the growth of pollen tubes. At 6 h after hand-pollination, the wild-type pollen tubes grew 917 μm on average, whereas those of *aif2-1* and *aif2-1*/*aif4-1* plants were longer and those of AIF2ox plants were shorter (Figures 2F,G). This retarded pollen growth in AIF2ox plants was also confirmed by the fact that all AIF2ox pollen tubes germinated *in vitro* were in the range of 0 to 150 μm (average 67.2 μm), whereas those of Col-0 and *aif2-1* plants grew 240 μm and 184 μm on average, respectively (**Supplementary Figure 4**). Nonetheless, most pollen tubes of Col-0 (1,468 μm), AIF2ox (914 μm), *aif2-1*(1,746 μm), and *aif2-1*/*aif4-1*(1,920 μm) reached almost the end of the pistils at 12 h, considering the pistil lengths of Col-0 (1.74 mm), AIF2ox (1.08 mm), *aif2-1* (1.92 mm), and *aif2-1*/*aif4-1* (2.53 mm) (Figure 2A). These results suggest that pollen tube growth is unlikely the reason for reduced male sterility and seed productivity in the AIF2ox plants. Collectively, we demonstrated that the defective silique growth and seed production in *AIF2*-overexpressing transgenic plants were caused by the reduced amount of pollen production and less-effective pollen tube germination but not by retarded stamen/pistil growth or pollen tube elongation.

### Expression Patterns of Pollen- and Auxin-Related Genes Were Significantly Modulated in AIF2-Overexpressing Plants

**FIGURE 3:**
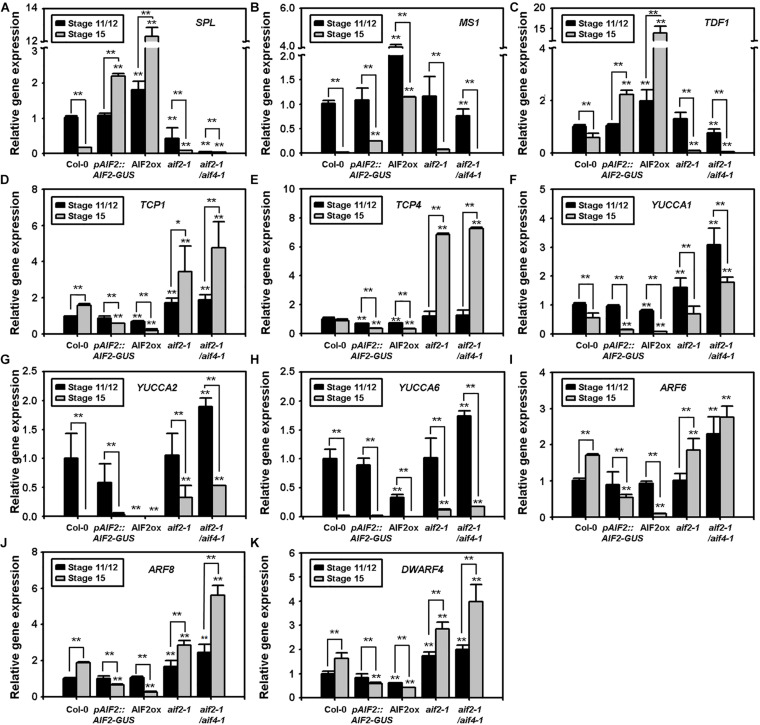
**(A–K)** Transcript expressions of pollen- and seed-regulating transcription factors **(A–C)**, auxin biosynthesis **(D–H)** and signalling-related genes **(I,J)**, and a brassinosteroid biosynthesis gene, *DWF4* **(K)**. Total RNA was isolated from flowers at floral stages 11/12 or 15, and transcript expression of genes was examined using qRT-PCR. Relative expression of each gene is normalised as a ratio of the Col-0 plants at floral stage 11/12, which was set to 1. Statistical difference from the same stage of the Col-0 flowers or bracketed samples is indicated by an asterisk (*) on bars at *P* < 0.05 and two asterisks (**) at *P* < 0.01.

Timely expression of *SPL*/*NZZ*, *TDF1*, and *MS1* is essential for early microspore mother cell formation to late pollen maturation (Yang et al., 2007; Ye et al., 2010; Chen L. G. et al., 2019; Chen W. et al., 2019). We examined transcript expression of these genes in flowers at floral stages 11/12 and 15. Two mitotic divisions of microspores and tapetum degeneration occur at floral stage 11, and desiccation of pollen grains followed by anther dehiscence occurs in flowers of floral stage 12 (Kim et al., 2001). Then, the flower opens and is self-pollinated during the stages 13 to 15. As expected, in floral stage 15 of Col-0 and the two *AIF2* knockout plants, these genes were transcriptionally down-regulated compared to the transcription of these genes in stage 11 or 12 flowers (Figures 3A–C). Unexpectedly, we found that *SPL* and *TDF1* at stage 15 maintained a higher expression both in AIF2ox and *pAIF2:AIF2-GUS* plants. In addition, although *MS1* in floral stage 15 showed lower expression than that at stage 11/12, a relatively higher expression was maintained than that of the same floral stage in Col-0 and the two *AIF2* knockout plants.

Auxin plays important roles in the proper development of flower and seeds (Shirley et al., 2019); thus, the mutants defective in auxin biosynthetic genes, such as *YUCCA*s, show not only abnormal flowers but also defects in the embryo and endosperm of seeds (Cheng and Zhao, 2007; Figueiredo and Köhler, 2018). TEOSINTE BRANCHED 1, CYCLODEA, and PROLIFERATING CELL FACTORSs (TCPs) bind to the promoters of *YUCCAs* to promote their gene expression and directly upregulate auxin levels (Challa et al., 2016; Zhou et al., 2018). Moreover, TCP1 promotes BR biosynthesis by directly upregulating the expression of a BR-biosynthetic gene *DWARF4* (*DWF4*) (Guo et al., 2010). We found that *TCP1* and *TCP4* genes of AIF2ox or the *pAIF2:AIF2-GUS* plants were relatively down-regulated at stage 11/12 compared with those of Col-0 plants at the same stage, and they were further downregulated at stage 15 (Figures 3D,E). Interestingly, these two genes were greatly upregulated at stage 15 and/or stage 11/12 of *aif2-1* and *aif2-1*/*aif4-1* plants. Similarly, three auxin biosynthetic genes (*YUCCA*s) and genes for two positive regulators of auxin signalling (*ARF*s) were down-regulated at either stage 11/12 (*YUCCA1*, *YUCCA2*, and *YUCCA6*) or 15 (*YUCCA1*, *YUCCA2*, *ARF6*, and *ARF8*) of AIF2ox plants while expression of these genes in the two *AIF2* knockout plants at the same stage was relatively up-regulated compared with those of Col-0 plants (Figures 3F–J). Transcript expression pattern of *DWF4* was also similar to that of the *TCP1*, so that it was relatively down-regulated when *TCP1* was suppressed at stage 15 of two *AIF2*-expressing transgenic lines (Figure 3K). These results indicate that aberrant expressions of pollen development-, auxin-, and BR-related genes in AIF2ox plants may partially explain the observed reduction in pollen grains together with the less-effective pollen tube germination and aborted seed development.

### AIF2ox Plants Differentially Regulated Transcript Expression of Seed-Forming Regulators

**FIGURE 4:**
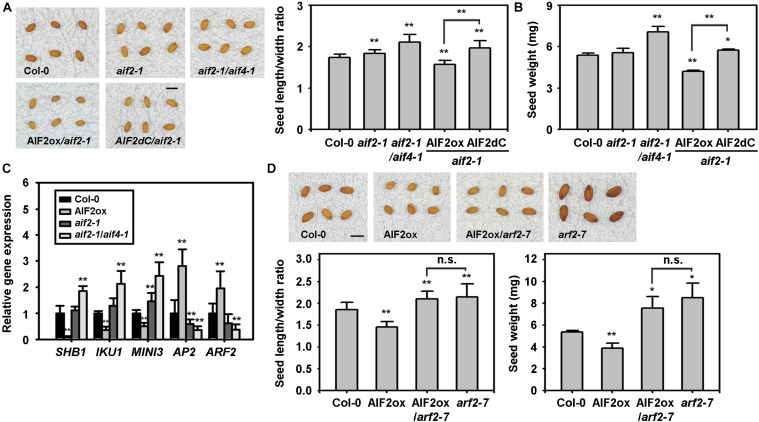
AIF2-mediated negative regulation of seed size and weight. **(A,B)** AIF2 overexpression-led smaller and rounder (less length to width ratio) **(A)** and lighter **(B)** seeds of *aif2-1* loss-of-function plants and their reciprocal confirmation through complementation assay with a dominant negative form of AIF2, AIF2dC. **(C)** Transcript expression of seed size-related genes in siliques with developing seeds. Relative expression of each gene is normalised as a ratio of the Col-0 plants, which was set to 1. **(D)** ARF2-dependent AIF2 effects on seed size and weight. Scale bars in pictures represent 0.5 mm in length. Statistical difference from the Col-0 control is indicated by an asterisk (*) at *P* < 0.05 and two asterisks (**) at *P* < 0.01. n.s., non-significant. Number of seeds examined for measurement of length/width ratio and weight >800.

Previously, BZR1-mediated BR signalling pathways were shown to increase seed size by affecting the integument, endosperm, and embryo development (Jiang et al., 2013). We found that ectopic expression of *AIF2* in *aif2-1* plants results in smaller and lighter plant seeds. The seed length to width ratio in Col-0 or *aif2-1* and *aif2-1*/*aif4-1* plants was 1.8–2.2, and ectopic expression of *AIF2* in the *aif2-1* plants modified the average ratio to 1.54 (Figure 4A). This implies that seeds of *AIF2*-overexpressing plants were likely to be rounder rather than ellipsoidal, typical of the seeds of Col-0 and the two *AIF2* knockout plants. As for a confirmation of the AIF2 functions in seed size and weight determination, we demonstrated that expression of C-terminal deleted *AIF2* (AIF2dC, a dominant negative form of AIF2 function, Kim et al., 2020) obliterated this complementation effect. In addition, AIF2ox plants produced lighter seeds than those of Col-0, *aif2-1*, *aif2-1*/*aif4-1*, and AIF2dC-overexpressing *aif2-1* plants (Figure 4B). Accordingly, we found that expression of the endosperm- and embryo-forming *SHB1*, *IKU1*, and *MINI3* were greatly reduced in AIF2ox plants. In contrast, *AP2* and *ARF2*, which negatively act in seed formation, were upregulated (Figure 4C) in the same AIF2ox plants. ARF2 is a transcriptional repressor of auxin-regulated genes, and *arf2* loss-of-function mutations increased seed size and weight as well as showed late flowering phenotypes under long day conditions in *Arabidopsis* (Schruff et al., 2006; Choi et al., 2018). To further investigate genetically whether the increased expression of *ARF2* in the AIF2ox plants was responsible for the small-seed phenotype, we crossed pollen of AIF2ox with ovules of *arf2-7* plants and found that an ectopic expression of AIF2 did not modulate *arf2-7* seed phenotypes (Figure 4D). These findings suggest that AIF2 acted upstream of ARF2 in negatively regulating seed shape and weight.

**FIGURE 5:**
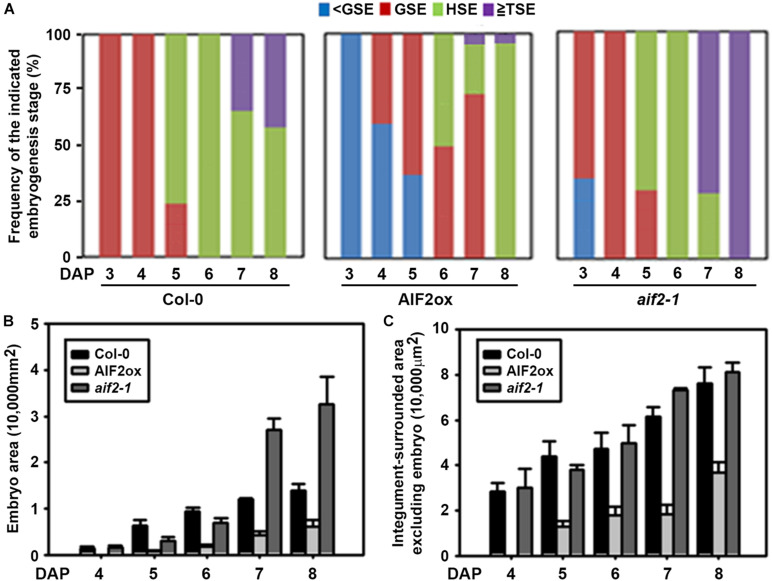
Delayed embryogenesis and smaller embryo development in *AIF2*-overexpressing *p35S:AIF2-EGFP* (AIF2ox) transgenic plants. **(A–C)** Comparison of the frequency of developmental stages of embryos **(A)**, size of embryo area **(B)**, and the size of remaining integument-surrounded area excluding the embryo **(C)** observed at different days after hand-pollinated self-pollination (DAP). <GSE, pre-globular stage; GSE, globular stage embryo; HSE, heart stage embryo; TSE, torpedo stage embryo. Number of embryos examined for each time point >150–200.

Reduction in seed size often results from coordinated reduction in endosperm size, embryo proliferation, and cell elongation of the maternally derived integument. *AIF2* was predicted to be highly expressed in the seed coats, chalazal endosperm, and spotted areas of peripheral endosperms through the pre-globular to torpedo stages (Supplementary Figure 5).^3^ In contrast, its expression was low in the developing embryo. To evaluate the effects of AIF2 on the endosperm- and embryo-forming processes, we morphologically investigated the progression of seed development in AIF2ox plants. All Col-0 plants at DAP3 progressed to globular embryos, whereas none of the AIF2ox plants showed distinct globular embryos (Figure 5A). At DAP6, all Col-0 and *aif2-1* plants developed into heart stage embryos. In contrast, almost half the AIF2ox plants remained as globular stage embryos. At DAP8, more than 40% of Col-0 plants had torpedo stage embryos, which further progressed in *aif2-1* plants such that all embryos were at the torpedo stage. Again, most embryos of AIF2ox plants were still at the heart stage, and only 5% of the total embryos were at the torpedo stage at DAP8. These results imply that embryonic progression is severely delayed in *AIF2-*overexpressing plants.

After fertilisation, the embryo grows to fill the cavity at the expense of the endosperm; thus, at maturity, the seed contains only a single layer of endosperm cells in *Arabidopsis* (Olsen, 2001; Sun X. et al., 2010). We found that the embryo area was increased in Col-0 plants (Figure 5B). A dramatic increase in the embryo size was especially observed at DAP5 when more than 75% of embryos progressed to the heart stage from the globular stage. The embryo area of *aif2-1* at DAP7 and 8 was much larger than that of Col-0 plants, mainly because most *aif2-1* embryos were in the torpedo stage. In comparison, the average embryo area in AIF2ox plants was much smaller, mainly because of their delayed embryonic progression. For example, embryos of AIF2ox seeds were at the globular or heart stage at DAP6 when all embryos of Col-0 or *aif2-1* seeds were at the heart stage. Similarly, integument-surrounded seed area excluding the embryo area showed a size reduction in AIF2ox plants (Figure 5C). Collectively, our results demonstrated that *AIF2*-overexpressing transgenic plants suppressed genes encoding positive factors (*SHB1*, *IKU1*, *MINI3*) of seed size determination but promoted gene expression for negative factors (*AP2* and *ARF2*), resulting in delayed embryogenesis and seeds with smaller size.

### AIF2-Regulation of Seed Shape and Weight Is Epistatic to Those by BZR1 and BIN2

**FIGURE 6:**
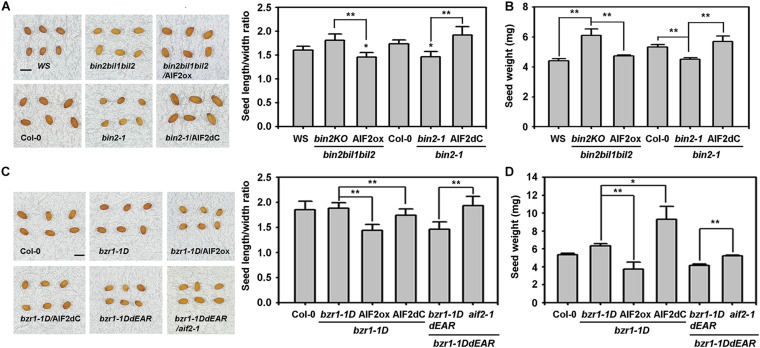
Genetic and functional interactions of AIF2 with BIN2 and BZR1 in the regulation of seed size and weight. **(A,B)** Evaluation of genetic and functional interaction of AIF2 with BIN2 in the regulation of seed size **(A)** and seed weight **(B)**. Transgenic constructs ectopically expressing AIF2ox or AIF2dC were transformed into a BIN2 gain-of-function mutant, *bin2-1*, or a BIN2 loss-of-function mutant, *bin2bil1bil*2 (*bin2*KO), and their seed phenotypes were evaluated. **(C,D)** Evaluation of genetic and functional interaction of AIF2 with BZR1 in the regulation of seed size **(C)** and seed weight **(D)**. Transgenic constructs ectopically expressing AIF2ox or AIF2dC were transformed into a BZR1 gain-of-function mutant, *bzr1-1D*, or plants constitutively expressing *bzr1-1DdEAR*, a loss-of-function form of BZR1, were crossed to *aif2-1* to examine their seed phenotypes. Statistical difference between bracketed samples is indicated by an asterisk (*) at *P* < 0.05 and two asterisks (**) at *P* < 0.01. Number of seeds examined for measurement of length/width ratio and weight >800. Scale bars in pictures represent 0.5 mm in length.

Previously, we demonstrated that AIF2 was significantly suppressed by BRI1/BZR1-mediated signalling pathways, and BIN2-mediated AIF2 phosphorylation augmented the BIN2/AIF2-mediated negative circuit of BR signalling pathways in growth-promoting cellular activities (Kim et al., 2017). In this study, *BIN2* triple knockout mutant (*bin2bil1bil2*, *bin2*KO) had ellipsoidal seed shape which was almost similar to that of WS plants (insignificant increase in seed length to width ratio) (Figure 6A). However, constitutive expression of *AIF2* in *bin2*KO background produced rounder seeds by significantly decreasing the seed length to width ratio. In contrast, *BIN2* gain-of-function mutant (*bin2-1*) produced rounder seeds, and an ectopic expression of a C-terminal deleted *AIF2* (*bin2-1*/AIF2dC) resulted in the ellipsoidal shape owing to an increase in seed length to width ratio. Similarly, transgenic expression of *AIF2* or C-terminal-deleted *AIF2* either decreased or increased seed weights in *bin2*KO and *bin2-1*, respectively (Figure 6B). These results suggest that AIF2 acted downstream of BIN2 in the regulation of seed size and weight.

Deletion of ERF-associated amphiphilic repression (EAR) motif at the carboxy terminus of BZR1 abolished the abilities to regulate gene expression and cell elongation (Oh et al., 2014). We found that transgenic expression of EAR-deleted *bzr1-1D* (*bzr1-1DdEAR*) produced round and light seeds, similar to the seeds of *bzr1-1D* that ectopically expressing AIF2ox. Again, expression of *AIF2dC* in *bzr1-1D* partially negated the AIF2 effects on seed shape determination (Figure 6C) and even greatly increased seed weights in the same plants (Figure 6D). These results imply that AIF2 acted downstream of BZR1 for seed size and weight determination. Supporting this idea, the described dominant negative effects of *bzr1-1DdEAR* in seed phenotypes were not functional in *aif2-1* genetic background plants. Thus, AIF2-controlled seed phenotypes acted downstream of BZR1 and BIN2, and BZR1-regulated seed shape and size were contrary to that by AIF2, whereas BIN2 functioned similar to AIF2.

### Transcript Suppression of Sucrose Transporter Genes and Lipid-Biosynthetic Genes in AIF2ox Plants and Subsequent Defects in Starch and Oil Accumulation

AIF2ox plants presented in this report not only delayed embryogenesis but also generated wrinkled and shrunken seeds (Supplementary Figure 6). Therefore, we examined starch accumulation in developing seeds, investigated transcript expression of proteins which promote sucrose transport and lipid biosynthesis, and scrutinised the cause of AIF2ox phenotypes.

**FIGURE 7:**
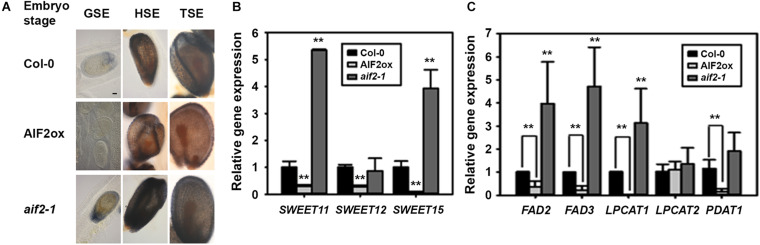
Reduced starch and oil accumulation in seeds of *AIF2*-overexpressing *p35S:AIF2-EGFP* (AIF2ox) transgenic plants. **(A)** Photographic comparison of starch accumulation in developing seeds. Siliques were stained with Lugol’s staining dye, and seeds with embryos at the same developmental stage were compared for starch accumulation. Number of seeds examined for each stage of the corresponding plants >150. Pictures show a representative image. **(B,C)** Total RNA was extracted from siliques with developing seeds, and the transcript expression for sucrose transport-related genes **(B)** and lipid biosynthesis genes **(C)** was measured using qRT-PCR. Relative expression of each gene is normalised as a ratio of the Col-0 plants, which was set to 1. Statistical difference from the Col-0 control is indicated by two asterisks (^∗∗^) at *P* < 0.01.

Starch is actively accumulated in the proliferating tissues, whole seed coat, ovary wall, placenta–septum region, and funiculus during early zygote and embryo development (Hedhly et al., 2016), which was also seen in the globular to torpedo stage embryos of Col-0 and *aif2-1* plants (Figure 7A). In contrast, starch granules in the seed coat of AIF2ox plants were relatively weakly stained with Lugol’s iodine dye. Sucrose, the major transport form of carbohydrate in plants, is delivered via the phloem to the maternal seed coat and then secreted from the seed coat to the embryo through SWEET11, 12, and 15 efflux carriers (Chen et al., 2015). Compared to the transcript levels of *SWEET11*, *SWEET12*, and *SWEET15* in Col-0, the transcript levels were greatly down-regulated in siliques of AIF2ox plants but upregulated in those of *aif2-1* (except for *SWEET12*) (Figure 7B). We hypothesised that reduced expression of sucrose transporter genes in AIF2ox plants and the subsequent defects in starch accumulation resulted in seeds with delayed embryogenesis and wrinkled phenotype.

Developing embryos of *Arabidopsis* and oilseed rape initially accumulated mother plant-driven starch, but the starch levels were declined with increase in the rates of storage lipid and protein synthesis (Andriotis et al., 2012). Accumulation of seed oil requires the co-ordination of *de novo* fatty acid (FA) biosynthesis and triacylglycerol (TAG) assembly. It was known that FA desaturase 2/3 (FAD 2/3), acyl-CoA:lysophosphatidylcholine acyl transferases (LPCATs), acyl-CoA:diacylglycerol acyltransferase 1 (DGAT1), and phospholipid:diacylglycerol acetyltransferase 1 (PDAT1) were positively involved in the modification of FAs and subsequent assembly of FA-driven acyl-CoA into glycerol, producing TAGs (Zhang et al., 2009; Xu et al., 2012; Lou et al., 2014). We showed that transcript expression of *FAD2*, *FAD3*, *LPCAT1*, and *PDAT1* was greatly suppressed in AIF2ox plants but promoted in *aif2-1* plants (Figure 7C). Thus, we concluded that suppressed expression of sucrose-transporting genes (*SWEET11/12/15*) and lipid-biosynthesis genes (*FAD2/3*, *LPCAT1*, and *PDAT1*) in *AIF2*-overexpressing transgenic plants resulted in reduced starch and lipid accumulation in the developing seeds resulting in shrunken and small phenotypes.

### Sucrose- and BR-Induced Repression of AIF2 Positively Controlled Seed and Silique Development

**FIGURE 8:**
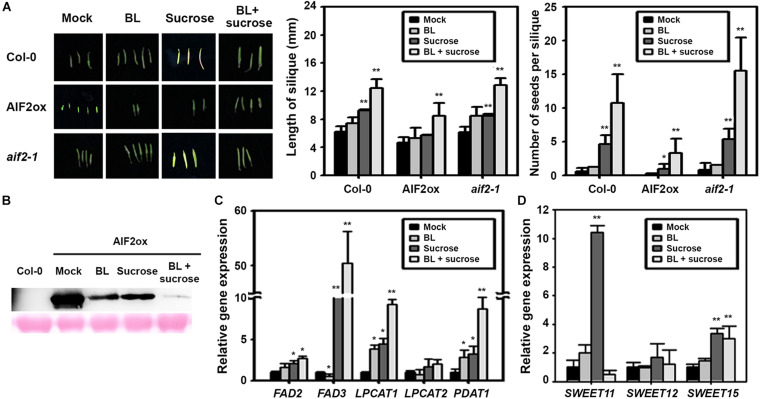
Sucrose- and brassinosteroid-induced suppression of AIF2 stability and silique phenotypes. Flowers in between DAP3.5 to DAP4 (*n* > 40 for each experiment) were collected and cultured for 9 days in solid MS media containing brassinolide (BL, 10^– 9^ M) supplemented with or without 3% sucrose (w/v). **(A)** Length and seed numbers of *in vitro*-cultured silique. **(B)** Western blot analysis of AIF2-EGFP expression in siliques of *in vitro*-cultured *p35S:AIF2-EGFP* transgenic lines (AIF2ox) **(C,D)** Total RNA was extracted from *in vitro*-cultured siliques of the AIF2ox, and the transcript expression for lipid biosynthesis genes **(C)** and sucrose transport-related genes **(D)** was measured using qRT-PCR. Relative expression of each gene is normalised as a ratio of the mock-treated plants, which was set to 1. Statistical difference from the mock-treated control is indicated by an asterisk (*) at *P* < 0.05 and two asterisks (**) at *P* < 0.01.

Sucrose is a necessary nutrient for embryo and seed development. Developing seeds form new carbon sink, generating high sugar flow from vegetative tissues to the seeds. To further confirm sucrose- and BL-mediated regulation of the AIF2ox phenotype, we examined the effects of BL and sucrose on silique phenotype and AIF2 stability in *in vitro*-cultured flowers. We found that the supply of BL did not result in an increase in silique length or seed numbers (Figure 8A). However, providing BL and sucrose promoted silique growth and seed production in AIF2ox, Col-0, and *aif2-1* plants, but was less effective in AIF2ox plants. Non-efficient promotion of BL itself might be attributed to the lack of nutrient supply found normally in intact plants. Such rescues of silique development were accompanied with a dramatic reduction in AIF2 stability in BL- and sucrose-treated AIF2ox plants (Figure 8B). BL-induced AIF2 degradation did not seem enough to cause the substantial recovery of silique growth because of the shortage in nutrients. Accordingly, supplying sucrose together with BL to the *in vitro* culture medium was the most effective in increasing transcript expression of *FAD3*, *LPCAT1*, and *PDAT1* (Figure 8C). Moreover, *SWEET15* was upregulated by the supplementation of BL or BL with sucrose (Figure 8D). Unexpectedly, *SWEET11* was highly upregulated by sucrose, and this effect was obliterated by the additional supplementation of BL. These results suggest that BR and sucrose reduced protein abundance of AIF2 transcription factor and increased starch and oil production for the successful generation of seeds.

## Discussion

BRs control diverse aspects of floral organ formation, seed development, and seed size determination. For example, two BR signalling activators, BES1 and its homologue BZR1, positively regulated tapetum and microspore development by directly upregulating *SPL/NZZ*, *TDF1*, and *MS1/2* (Ye et al., 2010; Chen L. G. et al., 2019; Chen W. et al., 2019). In contrast, the expression of *SPL* and *MS1* was significantly reduced in BR biosynthesis- (*cpd*) or signalling-defective (*bri1-116*, *bin2-1*) mutants producing greatly reduced number of pollen grains (Ye et al., 2010). Surprisingly, we found that *SPL* and *MS1* were highly upregulated in pollen- and seed-defective AIF2ox plants (Figures 3A,B). It is notable that *MS1*-overexpressing transgenic plants exhibited an excess deposition of wall materials and a loss of the regular structure of the pollen wall, eventually resulting in defective pollen development (Yang et al., 2007). MS1 protein was expressed in a developmentally regulated manner between late tetrad spore and microspore release and then broken down rapidly (Yang et al., 2007). Hence, it was suggested that MS1 breakdown was critical for the progression of pollen development, and the persistence of MS1 protein may serve to downregulate genes required for continued development of microspores. We showed that *SPL*, *MS1*, and *TDF1* in AIF2ox plants were relatively highly expressed even in flowers of stages 11/12 and 15 (Figures 3A–C). Moreover, AIF2ox plants showed retarded and defective progression of microsporogenesis, producing aberrant tetrad microspores (Figure 2E). Thus, it is possible that the stage-independent aberrant expression of pollen-producing genes such as *SPL* and *MS1* in AIF2ox plants may act reversibly on microspore development and viability.

**FIGURE 9:**
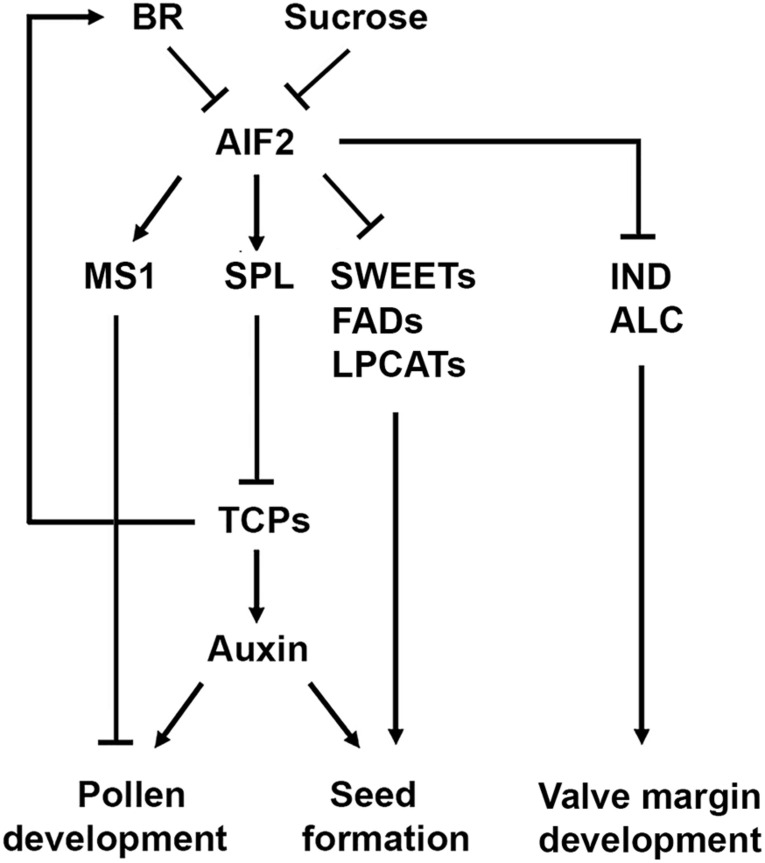
A diagram explaining AIF2-regulated pollen development and seed formation. Stage-independent aberrant expression of pollen-producing genes such as *SPL* and *MS1* together with suppression of sucrose-transporting *SWEET*s, lipid biosynthesis-regulating *FAD*s/*LPCAT*s, and valve margin-forming *IND*/*ALC* in AIF2ox plants may act adversely on pollen microspore development, auxin-regulated seed formation and proper silique development. Sucrose- and BR-induced repression of AIF2 bHLH transcription factor positively controls pollen development and/or seed size and numbers.

The auxin biosynthetic pathway is majorly regulated by catalytic activities of multiple monooxygenases encoded by the *YUCCA* genes, and TCP transcription factors can directly upregulate *YUCCA*s to increase auxin levels (Guo et al., 2010; Challa et al., 2016). Disruption of *TCP*s caused phenotypes that resemble *spl-D*, the heterozygous gain-of-function mutants of *SPL* (Wei et al., 2015). In other hand, *spl-D* mutants showed repressed expression of *YUCCA2* and *YUCCA6* and produced few and small flowers and short/wrinkled siliques with shrivelled seeds that could be partially rescued by crossing with *yuc6-D*, a dominant mutant of *YUC6* (Li et al., 2008). ARFs are a class of transcriptional modulators that regulate auxin-mediated gene expression. Likely, auxin biosynthesis-regulating genes, *Arabidopsis ARF6* and *ARF8*, through proper *microRNA167*-controlled cleavage, were critically involved in regulation of both gametophyte reproduction (Wu et al., 2006) and embryonic and seed development (Yao et al., 2019). In addition, ARF2 was negatively involved in the regulation of auxin-induced flowering time and seed size (Choi et al., 2018). Notably, similar to *aif2-1* plants, *arf2* loss-of-function mutants produced seeds with dramatically increased size and weight (Schruff et al., 2006). Based on the above studies, we suggest that ectopic expression of *SPL* in AIF2ox plants together with downregulation of *TCP*s, *YUCCA*s, and *ARF6/8* (Figure 3) and upregulation of *ARF2* (Figure 4C) may lead to the observed defective phenotypes of pollen, embryogenesis, and seeds/siliques (Figure 9). We demonstrated that AIF2-regulation of seed size and shape was epistatic to *bzr1-1D* and *bin2-1* genetic backgrounds (Figure 6). *bin2-1* exhibited reduced fertility, aborted ovules, and short siliques similar to those of AIF2ox plants, and auxin partially rescued the infertility phenotype of *bin2-1* (Li T. et al., 2019). Thus, it is probable that BIN2/AIF2 regulatory networks act via a coordinative interaction with auxin signalling pathways. In fact, rice OsSK41 (also known as OsGSK5, a BIN2 homologue) interacted with and phosphorylated OsARF4 (Hu et al., 2018). As a result, the expression of a common set of downstream genes was repressed, including some auxin-responsive genes during rice grain development; thus, the loss-of-function mutants of *OsSK41* and *OsARF4* showed increased grain length and weight. Further genetic analysis demonstrating *in vivo* functional interactions of BIN2/AIF2 and auxin signalling pathways are needed in future study.

Sucrose is delivered via the phloem to the maternal seed coat and then to the embryo through SWEET11, 12, and 15 efflux carriers (Chen et al., 2015). In addition, seeds with delayed embryogenesis and wrinkled phenotype commonly arise from defects in sucrose transport and endosperm formation (Andriotis et al., 2012; Chen et al., 2015). In this study, we further demonstrated that transcript suppression of sucrose transporter genes (*SWEET11*, *SWEET12*, and *SWEET15*) and lipid-biosynthesis genes (*FAD2*, *FAD3*, *LPCAT1*, and *PDAT1*) in *AIF2*-overexpressing plants resulted in the production of wrinkled seeds with reduced starch and oil levels (Figure 7). Similar to our *AIF2*-overexpressing transgenic plants, the *sweet11;12;15* triple mutant (lacking the ability to provide nutrients to the embryo and endosperm) showed delayed embryo development and reduced seed weight and lipid content, and exogenously supplied sucrose promoted embryo growth of *sweet11;12;15* mutants (Chen et al., 2015). *Arabidopsis SWEET*s such as *SWEET8* and *SWEET13* also played important roles in nurturing pollen grains; thus, mutation of these genes caused defective primexine deposition and pollen wall pattern formation resulting in male sterility (Sun et al., 2013).

Antisense expression of *CPD*, a gene involved in BR biosynthesis, in *Arabidopsis* impaired the ability of the plant to assimilate carbohydrates, and such transgenic plants displayed a clear reduction in starch content (Schlüter et al., 2002). Moreover, increasing BR levels in rice enhanced CO_2_ assimilation, favoured sucrose accumulation in the leaf, and increased assimilation of glucose to starch in the seed (Wu et al., 2008). Thus, the high expression level of *SPL* in AIF2ox plants and the subsequent reduction in *TCP1* expression followed by the decrease in BR biosynthesis resulted from a transcriptional decrease in *DWF4* may lead to attenuation of BZR1-mediated BR signalling pathways and reinforced BIN2/AIF2-mediated BR-defective signalling pathways. Supporting this idea, BR- and sucrose-regulated negative repression of AIF2 accumulation were co-related with accumulation of oil and starch and a resulting increase in seed number and silique length (Figure 8). Collectively, we propose that the impaired pollen and seed phenotypes of AIF2-overexpressing transgenic plants may be, in part, owing to the reduced capacity for sugar/starch production and defects in sugar transport during gametophyte formation, embryogenesis, and seed formation.

In this study, we demonstrated that multiple genes regulating development of pollen grains, seeds, and siliques were differentially regulated in AIF2ox plants (Figure 9). AIF2 is a non-DNA-binding bHLH transcription factor and it regulates target gene expression by binding to other DNA-binding bHLH proteins. Previously, we showed that AIF2 interacts with INDUCER OF CBF EXPRESSION 1 (ICE1), a nuclear-localised MYC-like bHLH transcription factor, via their C-termini (Kim et al., 2020). A successful formation of the AIF2–ICE1 complex, the subsequent direct upregulation of *C-REPEAT BINDING FACTORS* (*CBF*s), and the antagonistic downregulation of *PIF4* were negatively involved in dark-triggered and BR-induced leaf senescence, thus, helping plants continue to grow and remain green for a long time (Kim et al., 2020). Many transcription factors with bHLH domain have been shown to regulate flower and seed development. For instance, SPT can heterodimerise with ALC, and these two proteins apparently undergo sub-functionalisation with SPT, being essential for earlier development of carpel margin tissues, and ALC, specialising in later dehiscence zone development (Groszmann et al., 2011). Therefore, future studies need to verify whether AIF2 interacts with other bHLH family proteins and whether this interaction and the resulting functions depend on age-specific binding partners of AIF2 during plant reproductive processes.

## Data Availability Statement

All data associated with the paper are available in this manuscript. Novel materials used and described in the paper are available for non-commercial research purposes from the corresponding author (soohwan@yonsei.ac.kr).

## Author Contributions

Soo-HK managed whole experimental processes and wrote this manuscript. YK performed most experiments and generated Figures 2, 3, 5–9. Sun-HK made Figures 1 and 3. D-MS generated the *aif2-1*/*aif4-1* double knockout plants. All authors contributed to the article and approved the submitted version.

## Conflict of Interest

The authors declare that the research was conducted in the absence of any commercial or financial relationships that could be construed as a potential conflict of interest.

## Publisher’s Note

All claims expressed in this article are solely those of the authors and do not necessarily represent those of their affiliated organizations, or those of the publisher, the editors and the reviewers. Any product that may be evaluated in this article, or claim that may be made by its manufacturer, is not guaranteed or endorsed by the publisher.

## Footnotes

1
http://crispor.tefor.net/

2
http://rsbweb.nih.gov/ij/

3
http://bar.utoronto.ca/eplant/

## Supplementary Material

Supplementary Figure 1 Expression level-dependent aerial and silique phenotypes of AIF2-overexpressing independent transgenic lines (*p35S:AIF2-EGFP*/Col-0, AIF2ox).

Supplementary Figure 2 Expression of the native promoter-driven and CaMV35S promoter-driven AIF2 protein in floral organs.

Supplementary Figure 3 Reduced pollen production in AIF2ox plants.

Supplementary Figure 4 Efficacy of *in vitro*-germinated pollen tube growth.

Supplementary Figure 5 Relative expression of *AIF2* (At3g06590) in developing seeds.

Supplementary Figure 6 Morphology of seeds stained with mPS-PI, followed by confocal microscope observation.

Supplementary Table 1 Primers used in cDNA amplification of *AIF2*.

Supplementary Table 2 Primers used in quantitative real time RT-PCR analysis.

## Abbreviations

AIF2: ATBS1-INTERACTING FACTOR 2
AIF2ox: *AIF2* over-expressing *p35S,AIF2-EGFP* transgenic plants
bHLH: basic-helix-loop-helix
BL: brassinolide
BR: brassinosteroids
DAP: days after pollination.
