# Supplementary material for: ATBS1-INTERACTING FACTOR 2 Negatively Modulates Pollen Production and Seed Formation in Arabidopsis
Source: Front Plant Sci. 2021 Jul 27;12:704958. doi: 10.3389/fpls.2021.704958 (PMC8353282; doi:10.3389/fpls.2021.704958)
Supplement: Supplementary Figure 1 — Expression level-dependent aerial and silique phenotypes of AIF2-overexpressing independent transgenic lines (p35S:AIF2-EGFP/Col-0, AIF2ox). [file Data_Sheet_1.pdf]

Article title: ATBS1-INTERACTING FACTOR 2 negatively modulates pollen production and seed formation in *Arabidopsis*

Authors: Yoon Kim, Sun-Ho Kim, Dong-Min Shin, Soo-Hwan Kim

The following Supporting Information is available for this article:

Figure S1. Expression level-dependent aerial and silique phenotypes of AIF2-overexpressing independent transgenic lines (*p35S::AIF2-EGFP*/Col-0, AIF2ox).

Figure S2. Expression of the native promoter-driven and CaMV35S promoter-driven AIF2 protein in floral organs.

Figure S3. Reduced pollen production in AIF2ox plants.

Figure S4. Efficacy of *in vitro*-germinated pollen tube growth.

Figure S5. Relative expression of *AIF2* (At3g06590) in developing seeds.

Figure S6. Morphology of seeds stained with mPS-PI, followed by confocal microscope observation.

Table S1. Primers used in cDNA amplification of *AIF2*

Table S2. Primers used in quantitative real time RT-PCR analysis

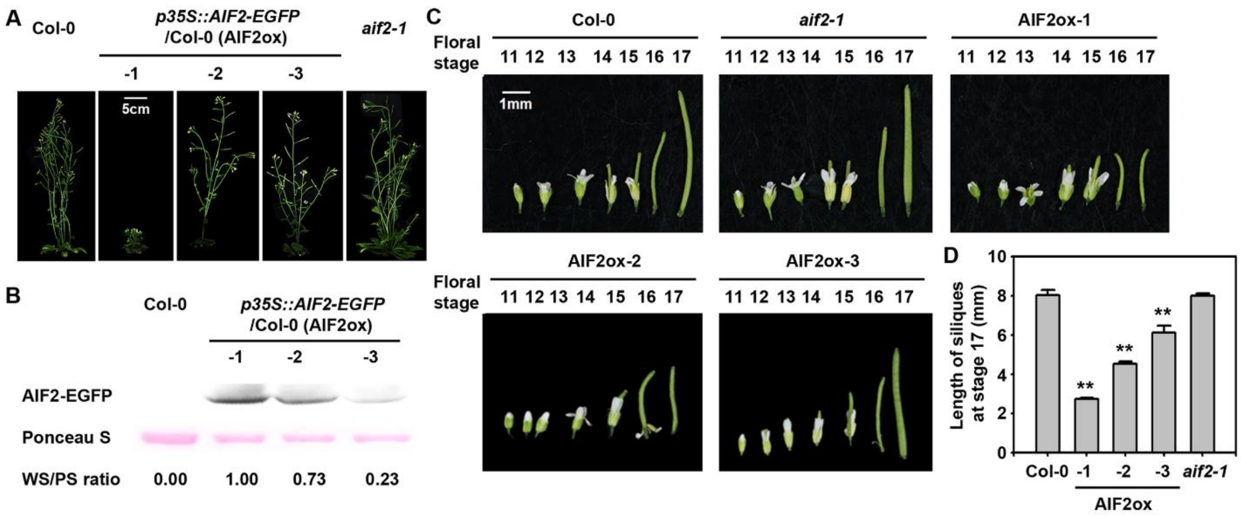

Supplementary Figure S1. Expression level-dependent aerial and silique phenotypes of AIF2-overexpressing independent transgenic lines (*p35S::AIF2-EGFP*/Col-0, AIF2ox). **(A)** Aerial phenotypes of 8-week-old plants. **(B)** Western blot analysis showing ectopic expression of AIF2-EGFP, ranging from high to low levels compared with the Columbia wild-type (Col-0) control. Total protein extracts were isolated from leaves of plants described in (A), size-fractionated by SDS-PAGE, and probed against anti-GFP antibodies. Ponceau S staining was used to normalize AIF2 expression level (WS/PS ratio). **(C, D)** Pictures showing floral stage-dependent growth of siliques (C) and measurement of silique length at stage 17 (D). Number of siliques examined > 50.

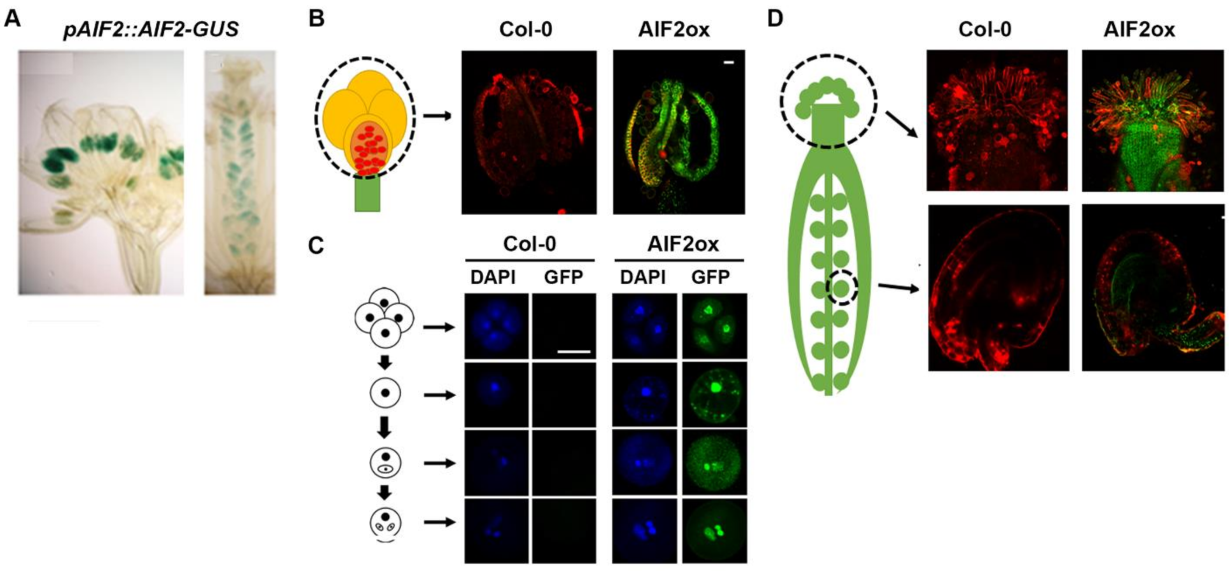

Supplementary Figure S2. Expression of the native promoter-driven and CaMV35S promoter-driven AIF2 protein in floral organs. **(A)** accumulation of the native promoter-driven AIF2-GUS protein in pollen grains and ovules **(B-D)** accumulation of ectopically-expressed AIF2-EGFP protein in anthers (B), developing pollens (C), and stigma/style and ovules (D). Bar represents 10μm in length. Red: PI-stained fluorescence, Blue: DAPI-stained fluorescence, Green: GFP fluorescence.

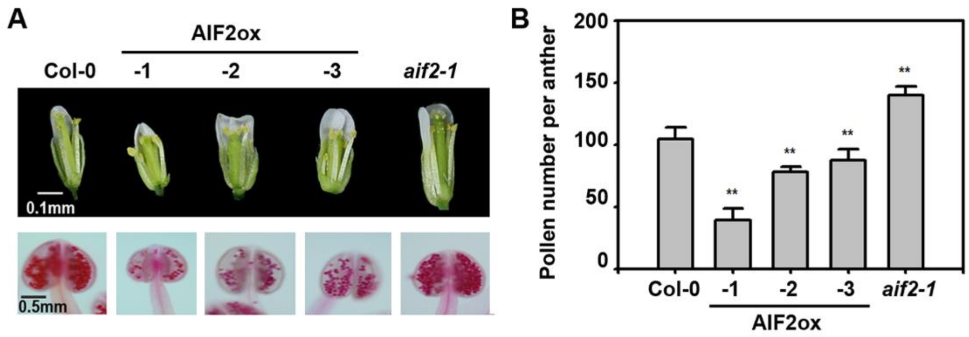

Supplementary Figure S3. Reduced pollen production in AIF2ox plants. **(A)** Anthers of different transgenic lines were stained with Alexander's solution and **(B)** the resulting dye-stained pollens were counted. Number of anthers examined > 50.

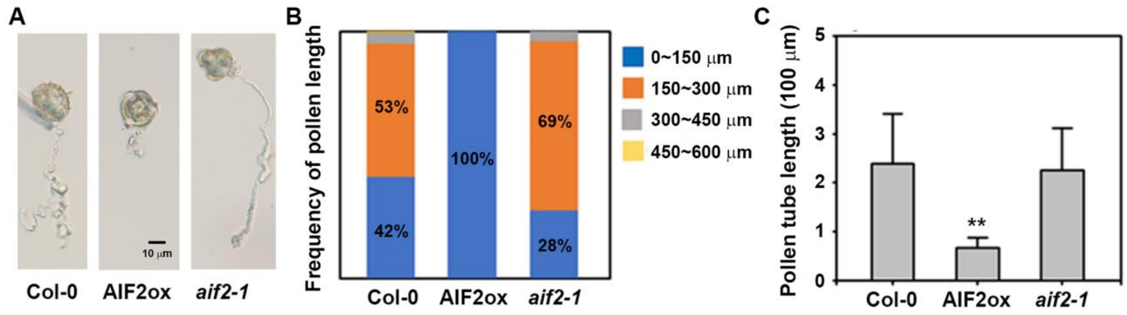

Supplementary Figure S4. Efficacy of *in vitro*-germinated pollen tube growth. Pollen grains of Col-0, AIF2ox, *aif2-1* plants were grown on a solid germination medium for 6 hrs in dark at room temperature. Data show **(A)** pictures of germinated pollen grains, **(B)** frequency of pollens that show tube growth in a range of the indicated length, and **(C)** the average tube length of all germinated pollens. Number of pollens examined > 3,000 taken from 20-25 open flowers.

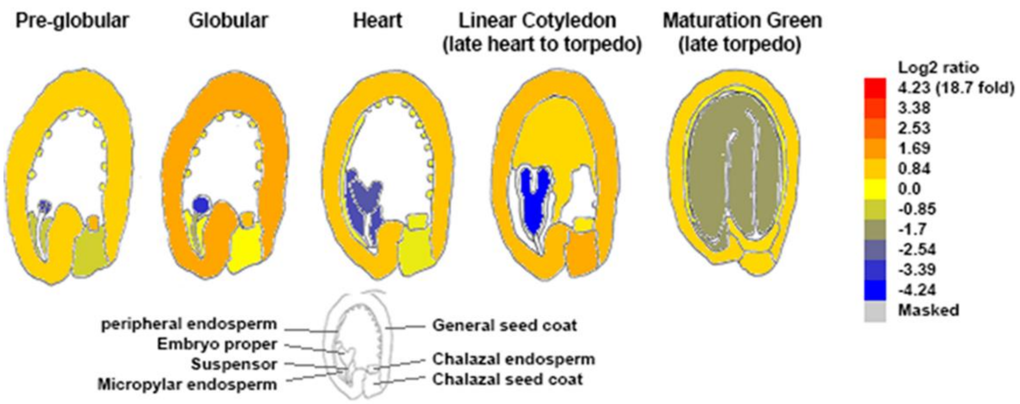

Supplementary Figure S5. Relative expression of *AIF2* (At3g06590) in developing seeds. Tissue-specific expression was analyzed using a plant eFP viewer tool provided by ePlant web site (http://bar.utoronto.ca/eplant/, University of Toronto).

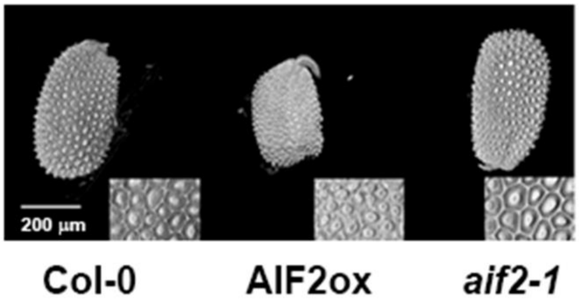

Supplementary Figure S6. Morphology of seeds stained with mPS-PI, followed by confocal microscope observation. The insert in picture is the magnified image of seed surface.

**Supplementary Table S1.** Primers used in cDNA amplification of *AIF2*		
Amplification	Gene fragment cloned	Primer sets (5' to 3')
AIF2FL	Full-length cDNA of *AIF2* coding region	CACCATGGCGTCTCTGATCTCAGATAT
		AATCGGTGGAGGAGCTGAGCCG
AIF2dC	cDNA encoding N-terminus and bHLH domain of *AIF2*	CACCATGGCGTCTCTGATCTCAGATAT
		CTCCAGAGCCTGAATATAATCAGTTGC

Supplementary Table S2. Primers used in quantitative real time RT-PCR analysis		
Gene	Locus	Primer set (5′ to 3′)
IND	At4g00120	CAGCCCCAAAAGAAGCATGATGG
		TTAGGGTTTCGGGAGGTGGATCTAA
ALC	At5g67110	CTCTCGAGCTTTCTCCGAACGATTC
		TGTCTTGGCCAGTTTCAGAGACTCC
MS1	At5g22260	TGGTGGGTGGTCAAATAGAG
		TCATCATTCCTACGTTCCCT
SPL	At4g27330	TCGCTAGAGCAGCTTCAGTT
		CCTCCATTGGTCCCGTAT
TDF1	At3g28470	CGGTTCCTCAAGTAGTGGG
		ATGTATTCGGCTTCGATGTT
TCP1	At1G67260	GGTACGGTGAAGAAGAAGTGG
		TCCTCTAGCTTTGGCTCCTAG
TCP4	At3G15030	AGGAGCAGAGCATCCGAGTA
		ATTGACGGCGGGAGAAAACT
YUCCA1	At4g32540	CTCGTCCGACATAACGCATCTCCT
		CCCAACCGGTGTATTTCCAAAC
YUCCA2	At4g13260	TCCCTAAAGATTTCTGTGAG
		CCACCGTGATACATACTCC
YUCCA6	At5g25620	GGGAGAAGAAGGCACGAC
		GAAGGCTGAGCACCGAAG
ARF6	At1g30330	GCATGCTGATGTGGAGACTGATGAA
		GGCTGCTGCGAGTAATCCAAGG
ARF8	At5g37020	TGGGTCAACAGGGTCATGAAGGAGA
		TGTGGTGGTAGGCTTGGGTAATTGG
DWF4	At1g78540	ACAGCAAAACAACGGAGCG
		TCTGAACCAGCACATAGCCT
SHB1	At1g78540	CAGGTTCAAGCACTGAGGAGT
		TGCTTCCTCGGTTTAGAGTA
IKU1	At2g35230	TTTGGGTGTGAATAGGATTGG
		CGCTGCAACCGTGTATTCT
MINI3	At1g55600	TTTGATGATATTGCAACGGAA
		GATCCTTTGTGTCTTGCTTGT
AP2	At4g36920	ATTCGGCTAATTCGAAGCATAA
		AGAGGAGGTTGGAAGCCATT

**Supplementary Table S2. Primers used in quantitative real time RT-PCR analysis**		
Gene	Locus	Primer set (5′ to 3′)
ARF2	At5g62000	TTTACCAGCAAGCGGACTTT
		CTGGCTGAGGACATCCAGTT
SWEET11	At3g48740	GGAGCCACACGTGCTAAGATTATCG
		ACCAAGCACATTCGGGAAAGCAA
SWEET12	At5g23660	GCGCCGCTTAGCATTATCAGAAC
		TGCACCTAGAACAAACCCAATCACG
SWEET15	At5g50800	CGTGGCTCGTGTGATAAAGACAAAG
		CCACCACGTTTGGAATCGCTATG
FAD2	At3g12120	TAACGTTATCGCCCCTACGTCAGC
		AATTGGTGGCGACGTAGTAGAAGCA
FAD3	At2g29980	CACTCGCGGTTCTTAAAGTCTACGG
		TGGCGTCGACCAAGTGATAGTGA
LPCAT1	At1g12640	GCAACAAGCGATCAGTCCGAAAATG
		AAGCGCGGGAAACTTACTTACCG
LPCAT2	At1g63050	TGCGGTTCAGATTCCGCTTTTCT
		TGTTGCCACCGGTAAATAGCTTTCG
PDAT1	At5g13640	TGCGAGCTTCCCTCAGTATGTAACG
		GCTGGATCCAACCCAGTTTCATTGT
UBC1	At1g14400	TCATCGCACGGTGATATTGAGAATC
		TCGACATCCTCCTTTCTTTCGTGGA
